# Supplementary material for: Comparison of Six Antifungal Susceptibilities of 11 Candida Species Using the VITEK2 AST–YS08 Card and Broth Microdilution Method
Source: Microbiol Spectr. 2022 Apr 6;10(2):e01253-21. doi: 10.1128/spectrum.01253-21 (PMC9045382; doi:10.1128/spectrum.01253-21)
Supplement: SUPPLEMENTAL FILE 1 — Supplemental material. Download SPECTRUM01253-21_Supp_1_seq5.pdf, PDF file, 0.2 MB [file spectrum01253-21_supp_1_seq5.pdf]

Supplementary Table 1. Flucytosine MIC distributions of tested isolates

			MIC (mg/L)										
Flucytosine	n	method	0.06	0.12	0.25	0.5	1	2	4	8	16	32	>64
Candida albicans	24	BMD	13	2			1		1				7
	24	YS08					16						8
Candida auris	45	BMD	11	19	13	2							
	45	YS08					44				1		
Candida glabrata	20	BMD	20										
	20	YS08					20						
Candida guilliermondii	9	BMD	8	1									
	9	YS08					9						
Candida krusei	15	BMD								4	10	1	
	15	YS08								3	11	1	
Candida lusitaniae	6	BMD	6										
	6	YS08					6						
Candida orthopsilosis	5	BMD	2	2		1							
	5	YS08					5						
Candida parapsilosis	19	BMD	12	5	2								
	19	YS08					19						
Candida pelliculosa	8	BMD	8										
	8	YS08					8						
Candida tropicalis	21	BMD	19	2									
	21	YS08					21						
Cyberlindnera fabianii	13	BMD	6	4	3								
	13	YS08					13						

* BMD, CLSI broth microdilution method; YS08, Vitek 2 AST YS08; n, number
